# Prevalence and Time Trend of SARS-CoV-2 Infection in Puducherry, India, August–October 2020

**DOI:** 10.3201/eid2702.204480

**Published:** 2021-02

**Authors:** Sitanshu Sekhar Kar, Sonali Sarkar, Sharan Murali, Rahul Dhodapkar, Noyal Mariya Joseph, Rakesh Aggarwal

**Affiliations:** Jawaharlal Institute of Postgraduate Medical Education and Research, Puducherry, India

**Keywords:** coronavirus disease, SARS-CoV-2, severe acute respiratory syndrome coronavirus 2, severe acute respiratory syndrome, SARS, viruses, respiratory infections, zoonoses, COVID-19, seroepidemiologic study, epidemiology, Puducherry, India

## Abstract

We conducted 3 population-based cross-sectional surveys, at 1-month intervals, to estimate the prevalence and time-trend of severe acute respiratory syndrome coronavirus 2 infection in Puducherry, India. Seropositivity rate increased from 4.9% to 34.5% over 2 months and was 20-fold higher than the number of diagnosed cases of infection.

The magnitude of the ongoing pandemic of coronavirus disease (COVID-19), caused by infection with severe acute respiratory syndrome coronavirus 2 (SARS-CoV-2), has not been fully assessed because most those infected have no or mild symptoms, and thus do not undergo viral nucleic acid or antigen testing (*1*–*3*). Determining the proportion of a population that has had infection at various time points is essential for understanding the dynamics of an epidemic in a particular area. 

Puducherry district, population ≈1.25 million, is located in southern India. Its earliest recorded case of COVID-19 was in March 2020; it had 7 total cases by the end of May, 67 by end of June, and 663 by end of July 2020 (*4*). The district followed national COVID-19 management guidelines, including testing all symptomatic persons and their high-risk contacts.

We conducted 3 community-based serologic surveys for SARS-CoV-2 antibodies in Puducherry at 1-month intervals, i.e., during August 11–16, September 10–16, and October 12–16, 2020 (Figure). Each survey included 900 adults selected using a multistage sampling procedure. In the initial stages, we chose 30 clusters, including 21 of 90 urban wards and 9 of 62 villages, using a probability proportional to size with replacement method; this method replicated the urban-to-rural ratio (70:30) of the district’s population. Thereafter, in each cluster, we chose 30 households by systematic random sampling; we collected blood from 1 adult (>18 years of age) in each household using a modified Kish method (*5*,*6*). The data from these surveys represent the cumulative proportion of population in Puducherry who had been infected with SARS-CoV-2 at ≈2 weeks before midpoint of each survey, i.e., at the end of July, August, and September 2020 (Figure). We obtained approval from Jawaharlal Institute’s ethics committee and informed written consent from participants.

**Figure:**
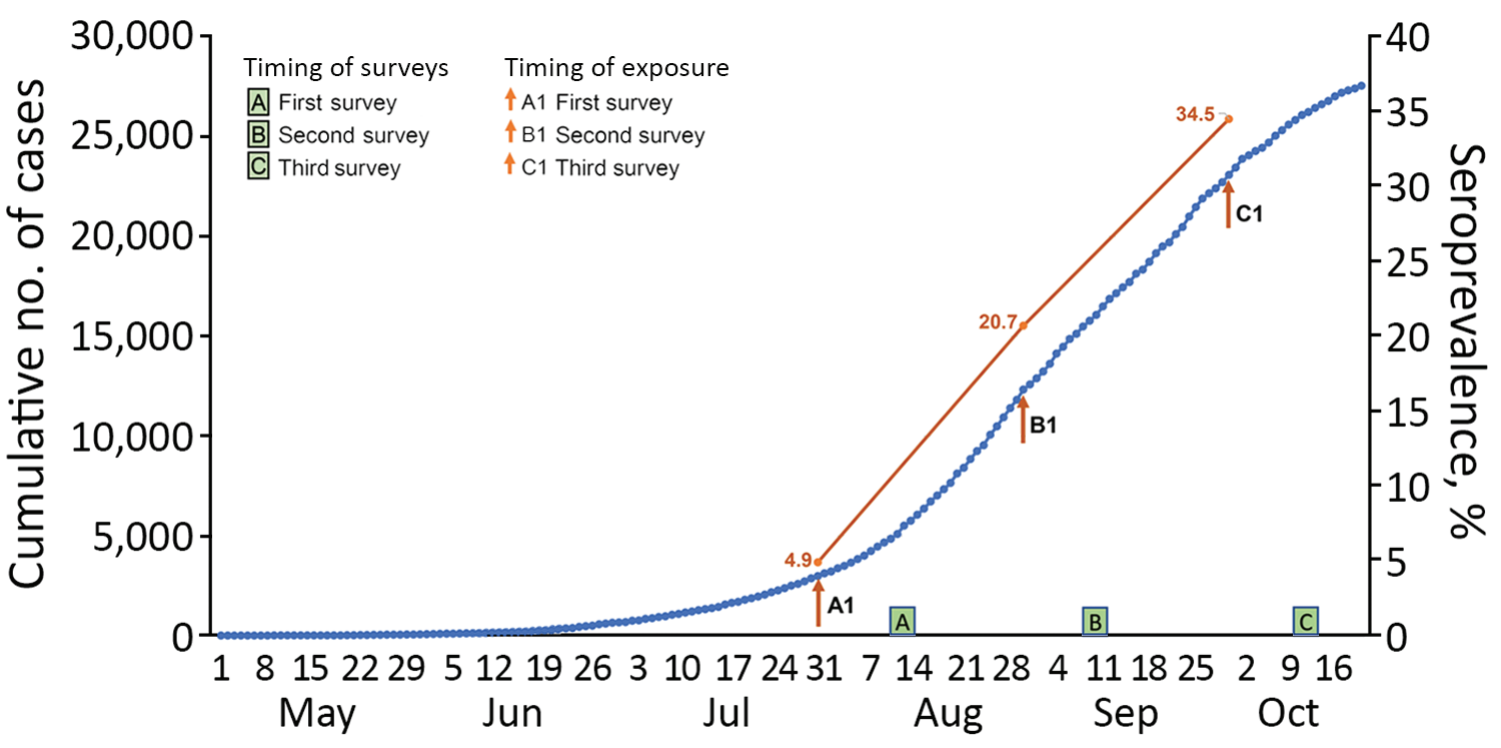
Prevalence of severe acute respiratory syndrome coronavirus 2 infection in 3 surveys in Puducherry district, India, 2020. Arrows indicate the timepoint 2 weeks before the midpoint of each of 3 surveillance periods.

We tested all serum specimens using a commercial electrochemiluminescence-based microparticle immunoassay with 99.5% sensitivity and 99.8% specificity (Elecsys Anti-SARS-CoV-2; Roche, https://www.roche.com) (*7*) for qualitative detection of antibodies against recombinant nucleoprotein antigen of SARS-CoV-2, following manufacturer’s instructions. Specimens with cutoff index >1.0 were considered seroreactive; cutoff index was the ratio of chemiluminescence signal of sample with that of the reference sample. For each timepoint, we calculated crude prevalence rate with 95% CI using a binomial model. In addition, we used the data on cumulative cases and deaths recorded until each timepoint (*4*) to calculate infection-to-case and infection-to-death ratios.

We visited 890 households and recruited 869 participants (response rate 97.8%) in August, 902 households from which we recruited 898 (99.8%) participants in September, and 900 households from which we recruited 900 (100%) participants in October. We tracked cumulative number of reported cases (cumulative incidence rates) of COVID-19 and deaths due to the disease in the district at each timepoint (Table) (*4*). In each survey, the median age was in the mid-40s with nearly equal numbers of men and women. Crude seroprevalence of SARS-CoV-2 antibodies increased from 4.9% (95% CI, 3.5%–6.4%) in August, to 20.7% (18.0%–23.3%) in September, to 34.5% (31.5%–37.7%) in October. These rates indicate that ≈16% of the district’s population acquired SARS-CoV-2 infection during August and ≈14% during September 2020. These rates are much higher than those reported from other parts of the world (*8*), but are similar to a high seropositivity rate of 57% reported in slum areas of Mumbai (*9*).

**Table:** Seroprevalence of SARS-CoV-2 antibodies in 3 surveys in Puducherry, India, 2020*

Variable	August 11–16, n = 869			September 10–16, n = 898			October 12–16, n = 900	
	No, positive/ no. tested	% (95% CI)		No. positive/ no. tested	% (95% CI)		No. positive/ no. tested	% (95% CI)
Crude prevalence	43/869	4.9 (3.5–6.4)		186/898	20.7 (18.0–23.3)		311/900	34.5 (31.5–37.7)
Age category, y								
18–29	8/170	4.7 (1.5–7.8)		33/165	20.0 (13.9–26.1)		58/180	32.2 (25.8–39.3)
30–44	13/295	4.4 (2.1–6.7)		58/277	20.9 (16.2–25.7)		92/252	36.5 (30.8–42.6)
45–59	13/242	5.4 (2.5–8.2)		64/271	23.6 (18.5–28.7)		101/259	39.0 (33.2–45.0)
>60	9/162	5.6 (2.0–9.1)		31/185	16.7 (11.4–22.1)		60/209	28.7 (23.0–35.1)
Sex								
M	16/439	3.6 (1.9–5.4)		95/443	21.4 (17.6–25.2)		126/406	31.0 (26.7–35.6)
F	27/428	6.3 (4.0–8.6)		91/455	20.0 (16.3–23.6)		183/491	37.2 (33.1–41.6)
Residence setting†								
Urban	35/609	5.7 (3.9–7.5)		130/629	20.7 (17.5–23.8)		225/628	35.8 (32.1–39.7)
Rural	8/260	3.1 (1.0–5.2)		56/269	20.8 (16.0–25.7)		86/272	31.6 (26.3–37.4)
Occupation‡								
Healthcare workers	2/29	6.9 (1.0–22.8)		4/32	12.5 (1.0–24.0)		18/66	27.2 (18.0–39.0)
Other frontline workers	0/22	0		8/23	34.8 (15.3–54.2)		6/15	40.0 (19.0–64.2)
Others	41/818	5.0 (3.5–6.5)		174/843	20.6 (17.9–23.4)		287/819	35.0 (31.8–38.3)
Other characteristics								
COVID-19	4/34	11.8 (9.3–22.6)		16/47	34.0 (20.5–47.6)		82/184	44.5 (37.5–51.7)
COVID-19 diagnosis	3/3	100		3/7	42.9 (6.1–79.5)		25/29	86.2 (69.4–94.5)
COVID-19 symptoms in last 6 mo	8/85	9.4 (3.2–15.6)		10/44	22.7 (10.3–35.1)		85/148	57.4 (49.3–65.1)
Cumulative case incidence (cumulative incidence ratio)§	2,987 (0.25%)			12,331 (1.03%)			23,080 (1.92%)	
Infection-to-case ratio¶	4.9%/0.25% = 19.6			20.9%/1.03% = 20.0			34.5%/1.92% = 18.0	
Cumulative deaths	43			187			441	
Infection fatality ratio (cumulative deaths per 100,000 infected persons)#	73.4			75.8			106.1	

*COVID-19, coronavirus disease; SARS-CoV-2, severe acute respiratory syndrome coronavirus 2. †Definitions used by the Office of the Registrar General & Census Commissioner, Government of India. ‡Other frontline workers included police officers, teachers, revenue officers, persons involved in COVID-19 response. §Calculated for data gathered until 2 weeks before the midpoint of the survey. ¶Infection-to-case ratio was calculated as crude seroprevalence / cumulative incidence ratio. #Infection-fatality ratio was calculated as cumulative deaths/crude prevalence × estimated population of the district.

The infection-to-case ratios were similar across the 3 surveys: 19.6 in August, 20.0 in September, and 18.0 in October. These results indicated that, despite implementing the strategies of testing all symptomatic persons and of aggressive contact tracing in the district, only a small proportion of SARS-CoV-2 infections had been diagnosed at each timepoint. This contrasts with the data from high-income countries (*10*) and could be related to the younger age distribution in the population of India, partial immunity due to other prior coronavirus or other infections, or both.

Strengths of our study include representativeness of the population by its random selection procedure and high participation rate; repeat testing in the same primary sampling units to reduce variability over time; and the use of an assay with high sensitivity and specificity. Limitations included the possibility that some persons did not show development of antibodies following infection, leading to a falsely low seroprevalence; possible loss of antibodies over time, leading to a falsely low rise of seroprevalence with time; and dependence of seroprevalence on the assay used.

Our data indicate a high rate of transmission of SARS-CoV-2 in Puducherry during August and September 2020, with some evidence of slowing over time. By the end of September, nearly one third of the population were infected with SARS-CoV-2, a much larger proportion than those diagnosed with COVID-19. These findings should help guide the response to COVID-19 in our district.
